# Effects of an intervention aimed at reducing the intake of sugar-sweetened beverages in primary school children: a controlled trial

**DOI:** 10.1186/s12966-014-0098-8

**Published:** 2014-07-25

**Authors:** Vivian M van de Gaar, Wilma Jansen, Amy van Grieken, Gerard JJM Borsboom, Stef Kremers, Hein Raat

**Affiliations:** 1 Department of Public Health, Erasmus University Medical Centre, Rotterdam, 3000, CA, the Netherlands; 2 Department of Social Development, City of Rotterdam, Rotterdam, 3000 BA, the Netherlands; 3 Department of Health Sciences, University Maastricht, Maastricht, MD, the Netherlands

**Keywords:** Overweight prevention, Sugar-sweetened beverages, Primary school children, Social marketing, Water, Integrated or community approach

## Abstract

**Background:**

Since sugar-sweetened beverages (SSB) may contribute to the development of overweight in children, effective interventions to reduce their consumption are needed. Here we evaluated the effect of a combined school- and community-based intervention aimed at reducing children’s SSB consumption by promoting the intake of water. Favourable intervention effects on children’s SSB consumption were hypothesized.

**Methods:**

In 2011-2012, a controlled trial was conducted among four primary schools, comprising 1288 children aged 6-12 years who lived in multi-ethnic, socially deprived neighbourhoods in Rotterdam, the Netherlands. Intervention schools adopted the ‘water campaign’, an intervention developed using social marketing. Control schools continued with their regular health promotion programme. Primary outcome was children’s SSB consumption, measured using parent and child questionnaires and through observations at school, both at baseline and after one year of intervention.

**Results:**

Significant positive intervention effects were found for average SSB consumption (B -0.19 litres, 95% CI -0.28;-0.10; parent report), average SSB servings (B -0.54 servings, 95% CI -0.82;-0.26; parent report) and bringing SSB to school (OR 0.51, 95% CI 0.36;0.72; observation report).

**Conclusions:**

This study supports the effectiveness of the water campaign intervention in reducing children’s SSB consumption. Further studies are needed to replicate our findings.

**Trial registration:**

Current Controlled Trials: NTR3400

## Background

Weight gain and subsequent overweight in children is a growing problem worldwide. One of the contributions to this problem is thought to be the consumption of sugar-sweetened beverages (SSB) [1]–[6]. Indeed, small reductions in daily SSB servings have been shown to potentially improve health [7],[8]; for example De Ruyter et al. showed that over a period of 18 months, children who replaced one SSB serving per school day with a non-caloric drink gained less weight, with an average difference of 1.0 kg [7]. Several other intervention studies with water as an alternative drink have demonstrated successful behavioural changes, weight loss and other health benefits [9]–[13].

As the number of obese children is not declining – at best it is levelling off [14]–[16] – effective interventions aimed at supporting a healthy lifestyle are needed. Schools are a relevant setting in which to improve healthy lifestyles among children, not only because most children attend school, but also because it allows an intervention to reach children with varied ethnicities and socio-economic backgrounds [17]–[26]. Nevertheless, schools are not the only setting that needs to be addressed. Parents also play an essential role in establishing healthy habits in children [24],[27]–[31]. In addition, the wider environment outside schools contains many so-called obesogenic determinants that should be targeted to promote a healthy environment. This means that interventions are needed at multiple levels [32]–[34]. As an example, Bleich et al. showed that community-based interventions that have a school component are more effective at preventing childhood overweight than interventions that are only school-based or only community-based [35]. Community involvement may also contribute to more sustainable programmes with higher reach and more impact [18],[32],[36]–[38]. It has therefore been suggested that childhood obesity must be addressed in multiple settings, i.e. at the individual, family, school and community level [17],[18],[24],[33],[35],[39],[40]. This advice with regard to multiple settings has led to initiatives such as the European EPODE network (where EPODE is a French acronym that stands for ’Together let’s prevent childhood obesity’) and its Dutch version JOGG, which are both using an approach that incorporates social marketing techniques [41]–[43]. These techniques are expected to enhance the outcomes of integrated approaches since many other social marketing-based programmes throughout the world have been successful [18],[36],[44]–[51].

Recently, within the JOGG city network in Rotterdam an intervention was developed aimed at reducing SSB intake. This water promotion intervention, called the ‘water campaign’, is school- and community-based and applies social marketing. The water campaign is an intervention tailored to children (aged 6-12 years) and their families who live in multi-ethnic, socially more deprived neighbourhoods; populations who remain disproportionately affected by childhood overweight [15],[52]–[54].

In this study, we evaluate the effectiveness of the water campaign. We hypothesized that after one year of intervention, children in the intervention group would have a lower SSB intake than children in the control group.

## Methods

### Intervention condition

The water campaign consists of lessons at school combined with integrated community activities that promote water consumption in various ways. Table 1 provides an overview of the water campaign activities. The intervention was developed by the local government using health promotion tools – intervention mapping [55] – in combination with social marketing. According to French et al., social marketing aims to change voluntary behaviour by taking the needs and wishes of the target audience as the starting point and from there trying to understand how best to promote the desired behaviour using an integrated, tailored approach [43].

**Table 1:** Overview of activities in the water campaign and regular health promotion programme

**Activities**	**Aimed at**	**Water campaign**	**Regular programme**
Three physical education lessons per week by professional physical education teacher	Children	□	□
School sport clubs	Children	□	□
Education in choosing healthy food and sports	Children & parents	□	□
School dietician	Children & parents	□	□
Annual height and weight measurements (for BMI tracking) and fittest	Children & parents	□	□
Additional non-compulsory play and sports activities outside school hours	Children	□	□
Special event: water campaign kick-off ‘Drinking water is fun!’	Children & parents	□	
Use of promotional material: posters ‘*Water is the best thing I can give to my child!’*	Children & parents	□	
Activity	Children & parents	□	
*For children:* Pimp up your water bottle		□	
*For parents:* Pimp up your water jug		□	
Provision of free water bottles by community organizations during summer activities	Children	□	
Provision of free water at school throughout the day	Children	□	
Taking a water break during physical education lessons; parents responsible for giving the child his/her water bottle to school	Children & parents	□	
Water theme week, including activities	Children & parents	□	
*For children:* special educational water lessons, fun games such as happy families, board and card games involving water consumption, and a special water show provided by children’s role models			
*For parents:* storytelling about promoting water consumption, different fun games involving water consumption and other aspects of water, including a water magazine for mothers; and promotion by water ambassadors			

Following the social marketing guidelines, desk research and focus-group interviews were applied to identify specific risk groups and risk behaviours. Based on these results, the local government intervention-development team decided to focus the water campaign on Turkish and Moroccan families [52]. These families form a large group of non-Western immigrants in the study area, a group disproportionately affected by childhood overweight [15],[53],[54]. Although the intervention was tailored to, pre-tested in and developed for children and mothers from these ethnic minorities, the water campaign was delivered to all children (and their families) attending the intervention schools and/or living in these neighbourhoods. By encouraging the children to consume more water, the water campaign intended to reduce children’s SSB intake [5],[7],[8],[10]–[13],[20],[23].

### Control condition

The intervention and control schools continued with their regular health promotion programme, the effective school-based curriculum ‘Enjoy Being Fit’ (EBF). Initiated in 2006, this multi-component programme for primary school children encourages a healthy lifestyle by educating children and providing additional extracurricular physical activity lessons (see Table 1) [56]. This programme addresses behavioural and environmental determinants based on elements of the ‘Environmental Research framework for weight Gain prevention’ [33]. A more detailed description of the EBF programme and a study describing its effects is provided elsewhere [57].

### Study design

To evaluate the water campaign, we conducted a controlled trial with baseline measures collected prior to the intervention and follow-up measures after one year of intervention. A controlled design was chosen for practical reasons that were related to the spread of intervention activities throughout the community. Four schools were included, which were randomly allocated to either intervention or control condition. The Medical and Ethical Review Committee of the Erasmus Medical Centre issued a ‘declaration of no objection’ (i.e. formal waver) for this study (reference number MEC-2011-183). Parents and children were informed about the study and were free to refuse participation without giving any explanation.

The outcome measure was children’s SSB consumption, which was estimated by means of parent and child questionnaires and observations of drinks brought to school. Blinding of participants and data collectors was not possible since the water campaign’s activities were visible at the intervention schools and throughout the neighbourhoods.

### Sample and participants

**Figure 1:**
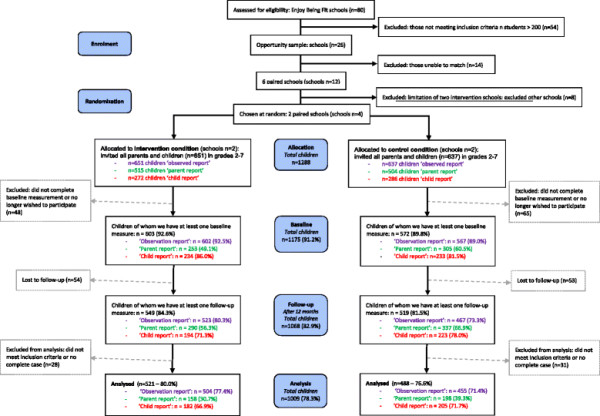
Overview of the course of the study.

Two intervention schools were assigned to the water campaign. A total of four schools, located in four different non adjacent neighbourhoods in Rotterdam, the Netherlands, were matched in pairs of two. The matched schools had a roughly equal number of pupils, had pupils of similar socio-economic status, and had a similar prevalence of overweight. On the basis of these criteria, we were able to select only six school pairs from the 80 schools that were eligible for the study. These pairs were then approached based on convenience, in the knowledge that a school pair could only be included in the study if both schools in the pair provided consent and a maximum of two school pairs in total could be included in the study. One of the schools in the school pair was then allocated to either the intervention or control condition by the flip of a coin. Figure 1 provides an overview of study enrolment and participant flow. At the four participating schools, all children in grades 2 to 7 (aged 6-12 years) were invited to participate, as were their parents.

### Power

The study was powered to detect a difference in SSB servings of 0.50 per day between the intervention and control groups. We hereby assumed a standard deviation of 1.00 serving, with a power of 0.80 and an alpha of 0.05 (two-sided), taking into account adjustment of baseline values and loss to follow-up (assuming a correlation of 0.80 between baseline and follow-up measurements).

### Measurements

Data on children’s SSB consumption and socio-demographic characteristics were collected at baseline and after one year of intervention, using parent and child questionnaires (assessed separately) and observations at school.

The parents of all children in grades 3 to 7 (aged 6-12 years) received the questionnaire at two time points: at baseline (April 2011) and after one year of intervention (June 2012). Children in grades 5 to 7 (aged 9-12 years) were invited to complete child questionnaires at two time points: at baseline (April 2011-September 2011) and after one year of intervention (June 2012). The observations of children in grades 2 to 7 took place at two time points: at baseline (April 2011-September 2011) and after one year of intervention (June 2012).

**Figure 2:**
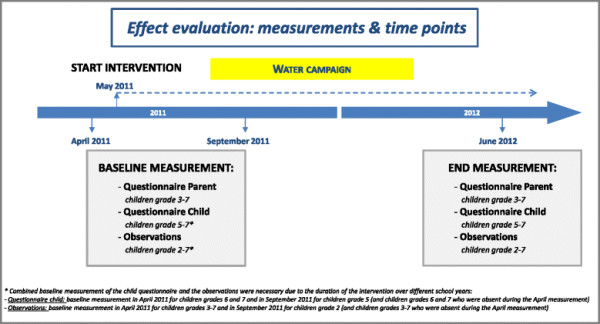
Overview of the data collection.

Since the intervention was implemented over two school years, it was necessary to combine the baseline measurements for the child questionnaire and the observations. Children in grades 6 and 7 completed the baseline questionnaire in April 2011; children in grade 5 (and children in grades 6 and 7 who were absent during the April measurement) completed their baseline questionnaire in September 2011. Children in grades 3-7 were observed for baseline measurement in April 2011 and children in grade 2 (and children grades 3-7 who were absent during the April measurement) were observed for baseline measurement in September 2011. Hypothetically, the fact that we used a combined baseline could have led to underestimation of effect because some children had already been exposed to the intervention. However, when we repeated the analyses using only the April 2011 data as baseline measurement we found similar results (data not shown). An overview of the data collection is presented in Figure 2.

### SSB consumption

The following definition of SSB was used: beverages containing added sugar, sweetened dairy products (e.g. chocolate milk), fruit juice (e.g. apple juice), soft drinks (e.g. cola) and energy drinks (e.g. sport energy drinks).

The consumption of SSB was assessed using similar questionnaire items for both parents and children (an overview of several items used in the parent and child questionnaire to assess child’s SSB intake are shown in Additional file 1: Table S1). Examples of SSB were provided, based on our definition of SSB. First, we asked whether the child consumed SSB on a daily basis. Answer categories were ‘yes, every day’; ‘no, not every day’; and ‘never’, except for the baseline parent questionnaire, where the answer categories were only ‘yes, every day’ or ‘no, not every day’. At follow-up, this outcome measure was recoded into ‘yes, every day’ and ‘no, not every day’ (including ‘never’).

Average SSB intake was measured by asking the child or parent to indicate how many glasses (250 ml), cans (330 ml) or bottles (500 ml) the child consumed on an average day on which the child drank SSB. Answer categories ranged from ‘none’ to ‘5 or more’. The total SSB intake per day, converted to litres, was calculated by summing up the multiplications of the number of glasses, cans or bottles with their volume. The average number of SSB servings was measured using the same question, adding up the number of glasses, cans or bottles that were reported to be consumed (under the assumption that a child would not consume multiple SSB drinks at once, e.g. consuming a glass and a can SSB simultaneously).

Observations at school were conducted by trained observers, who objectively recorded one morning on a random school day the drinks that the children brought to consume at school during morning break (10:00 am). Before analysis, the beverages were classified as ‘SSB’ or ‘not SSB’ based on the definition provided above.

### Socio-demographic factors

The parent and child questionnaires included items on child’s gender, age, grade and ethnic background. Ethnic background was determined by the country of birth of the parents according to definitions given by Statistics Netherlands [58]. The child’s ethnic background was defined as Dutch only if both parents had been born in the Netherlands; if one of the parents had been born in another country, ethnic background was defined according to that country; and if both parents had been born in different foreign countries, ethnic background was defined as the mother’s country of birth. Ethnic background was categorized as either Dutch, Surinamese/Antillean, Moroccan/Turkish or other/unknown.

Gender, age and educational level of the caregiver were also recorded using the parent questionnaire. The caregiver’s highest educational level was categorized as either ‘high’ (high/mid-high); ‘low’ (mid-low/low); or ‘unknown’, based on standard Dutch cut-off points [59].

### Weight status

Trained personnel measured height and weight at baseline. Weight status was determined by calculating the Body Mass Index (BMI) in kg/m^2^ with height measured to the nearest 0.1 cm and weight measured to the nearest 0.2 kg, in light clothing or gym clothes, according to a national standardized protocol for youth health care taking into account the child’s age and gender [60]. Children were categorized as being either ‘non-overweight’ or ‘overweight/obese’, based on cut-off points published by the International Obesity Task Force [61].

### Data analysis

To evaluate the water campaign’s effects on SSB consumption, we used the following three data sets: (1) data collected using parent questionnaires, from now on referred to as the ‘parent report’; (2) data collected using child questionnaires, from now on referred to as the ‘child report’; and (3) data collected using observations, from now on referred to as the ‘observation report’. All statistical analyses were performed using SPSS, version 21.0 (IBM Corp., NY, USA).

In all data sets, outliers were checked and implausible recordings were recoded as missing. For children lost to follow-up, we performed additional analyses that compared their data with that of children for whom follow-up data was complete. T-tests and Pearson Chi-square tests were used for comparisons at baseline.

To evaluate the intervention’s effectiveness, regression analyses were applied with a significance level of p < 0.05. Multi-level analyses were not possible due to the low number of clusters (i.e. four schools) [62]. Only complete case analyses were performed, meaning we analysed data only from children whose data from both time points was complete. The dependent variable was defined as the SSB measurement after one year of intervention. This meant that for the ‘parent report’ and ‘child report’ the outcome measures were ‘daily SSB consumption (yes/no)’ and ‘average SSB intake (in litres and number of servings)’; and for the ‘observation report’ the outcome measure was ‘daily SSB intake (yes/no)’. The condition (intervention/control) was entered as the independent variable. In all analyses, outcome measures were adjusted for baseline SSB values, several socio-demographic characteristics (grade, gender and ethnic background of the child and educational level of the caregiver) and child’s weight status at baseline. This was done by also entering them as independent variables. For the ‘parent report’, the caregiver’s age and gender were added to the analyses as a potential confounder if these variables differed at baseline between the intervention and control group. No imputations were performed for these potential confounders given the relatively small number of missing data points (range n = 3-35). Additionally, the analyses were corrected with the variable ‘school pair’ to adjust for the matching of schools. We explored interaction effects of ‘condition’ on the socio-demographic variables, child’s weight status at baseline and school pair (p < 0.10) [63].

## Results

In total, 1288 children were invited to participate in this study. At baseline, response was 54.8% among parents, 83.7% among children, and 90.8% for the observations. At follow-up, response was 61.5% among parents, 74.7% among children, and 76.9% for the observations.

We were able to conduct non-response analyses for the variables gender, grade and ethnic background of the child. Parents of children who participated in the study were more often parents of children in the lower grades (p < 0.001) and of children with a Dutch ethnic background (p < 0.001) as compared to parents lost to follow-up. Children who completed a questionnaire were more often children in the lower grades (p < 0.001) and children with a Dutch ethnic background (p = 0.007) as compared to children lost to follow-up. Children who were observed were more often children with a non-Dutch ethnic background (p < 0.001) as compared to children lost to follow-up.

Non-response analyses were also conducted for the condition variable. Here we saw a difference between the intervention and control conditions in parents of children who participated in the study compared to parents lost to follow-up (p = 0.006) and for children who underwent observation compared to children lost to follow-up (p = 0.014).

As shown in the flowchart depicted in Figure 1, the population for analysis comprised of 356 children using the data from the ‘parent report’ (34.9%); of 387 children using the data from the ‘child report’ (69.4%); and of 959 children using the data from the ‘observation report’ (74.5%). This meant that at least one complete case analysis could be performed for 1009 children (78.3%). In Additional file 1: Figure S1, a diagram is depicted to provide information on the combinations of responses between the three data reports.

### Baseline characteristics

Baseline measures of child and caregiver characteristics in both conditions are shown in Table 2. Children in the intervention condition were more often children in the higher grades (‘observation report’ p < 0.016), more often children with a non-Dutch ethnic background (‘parent report’ p = 0.033; ‘child report’ p = 0.001; ‘observation report’ p < 0.001), more often children of caregiver with lower educational levels (‘child report’ p = 0.001; ‘observation report’ p < 0.001), and more often children of younger caregivers (‘parent report’ p < 0.001) as compared to children in the control condition.

**Table 2:** Child and caregiver characteristics at baseline, in intervention and control group (n = 1288)

	**Parent report**^**a**^**(children grades 3-7)**			**Child report**^**a**^**(children grades 5-7)**			**Observation report**^**a**^**(children grades 2-7)**		
	***n*** **= 356**			***n*** **= 387**			***n*** **= 959**		
**Variable**	**Control (*n*** **= 198, 55.6%)**	**Intervention (*n*** **= 158, 44.4%)**	***P*** **value**^**b**^	**Control (*n*** **= 205, 53.0%)**	**Intervention (*n*** **= 182, 47.0%)**	***P*** **value**^**b**^	**Control (*n*** **= 455, 47.4%)**	**Intervention (*n*** **= 504, 52.6%)**	***P*** **value**^**b**^
*CHILD*									
Gender	57.7	55.1	0.625	55.0	50.6	0.386	52.9	52.2	0.826
% female									
Grades			0.349			0.250			**0.016**
% Grade 2	-	-		-	-		17.0	20.0	
% Grade 3	21.5	20.3		-	-		17.0	18.8	
% Grade 4	21.5	22.2		-	-		17.0	20.4	
% Grade 5	20.5	16.5		27.3	34.6		15.6	13.7	
% Grade 6	20.0	17.1		34.6	29.7		16.1	13.8	
% Grade 7	16.4	24.1		38.0	35.7		17.2	13.1	
Ethnic background			**0.033**			**0.001**			**<0.001**
% Dutch	29.3	41.1		17.6	29.7		24.2	35.5	
% Surinamese/Antillean	23.2	13.3		29.3	13.7		29.2	14.7	
% Moroccan/Turkish	30.8	27.2		33.2	32.4		27.3	32.9	
% Other/missing	16.7	18.4		20.0	24.2		19.3	16.9	
Weight status	24.5	22.1	0.600	26.7	24.3	0.600	25.1	21.6	0.207
% overweight or obese									
*CAREGIVER – if known*									
Gender	82.5	88.7	0.109	-	-		-	-	
% female									
Age (in years), mean (SD)	36.82 (6.60)	39.86 (6.62)	**<0.001**	-	-		-	-	
Educational level^c^			0.686			**0.001**			**<0.001**
% Unknown	2.5	2.5		20.0	33.0		24.4	37.1	
% Low	47.0	42.4		46.8	29.7		37.8	25.2	
% High	50.5	55.1		33.2	37.4		37.8	37.7	

^a^ = participants with complete data available at baseline and after one year of intervention; ^b^ = differences between intervention condition and control condition, as measured at baseline, tested with independent-samples *t*-test (continuous variables) and Chi-square test (categorical variables); ^c^ = caregiver’s educational level presented in the ‘child report’ and ‘observational report’ are based on data from the ‘parent report’. Note: numbers printed bold indicate significant *P* values.

### Intervention effects

Table 3 describes child’s SSB consumption at baseline, with only the ‘observation report’ showing the frequency of SSB being brought to school to be significantly lower in the intervention group than in the control group (p < 0.001).

**Table 3:** SSB consumption in outcome measures of the intervention and control groups

		**Intervention**		**Control**		**Effect of intervention**^**c**^					
						**Unadjusted effects**			**Adjusted effects**		
**Variable**	**N**	**Baseline**	**Follow-up**	**Baseline**	**Follow-up**	**B**	**OR**	**95%CI**	**B**	**OR**	**95%CI**
*Parent report*											
% SSB every day	312	57.2%	49.3%	60.2%	56.0%		0.75	0.46 - 1.24		0.79	0.47 - 1.34
Average SSB (L), mean (SD)	322	0.76 (0.56)	0.66 (0.41)	0.85 (0.54)	0.84 (0.43)	**−0.16**^**a**^		−0.24 - -0.07	**−0.19**^**a**^		−0.28 - -0.10
Average SSB servings (#), mean (SD)	322	2.74 (1.68)	2.39 (1.28)	3.05 (1.61)	2.92 (1.34)	**−0.42**^**a**^		−0.69 - -0.15	**−0.54**^**a**^		−0.82 - -0.26
*Child report*											
% SSB every day	350	24.4%	37.5%	26.2%	32.3%		1.33	0.86 – 2.07		1.32	0.78 – 2.24
Average SSB (L), mean (SD)	365	1.31 (0.85)	1.16 (0.75)	1.49 (0.92)	1.15 (0.64)	0.06		−0.07 – 0.20	0.04		−0.10 – 0.19
Average SSB servings (#), mean (SD)	365	4.09 (2.33)	3.70 (2.10)	4.53 (2.54)	3.70 (1.88)	0.16		−0.23 – 0.54	0.05		−0.36 – 0.47
*Observation report*											
% SSB brought to school	902	68.7%^b^	68.8%	79.6%^b^	82.2%		**0.51**^**a**^	0.37 - 0.70		**0.51**^**a**^	0.36 - 0.72

Note: Mean values (SD) of average SSB intake in litres, number of servings, and prevalence of children taking SSB with them to school. Figures are given for baseline and follow-up, in intervention and control groups. Also shown is the intervention effect (B (95% CI) and OR (95% CI)) between both groups for the parent reports (n = 356), child reports (n = 387) and observation reports (n = 959). Significant effects are shown in bold. ^a^ = in favour of intervention group; ^b^ = difference (p < 0.05) between intervention and control group at baseline; ^c^ = regression coefficients and odds ratios of the unadjusted models (only adjusted for baseline intake and school-pair) and fully adjusted models (in addition to baseline intake and school-pair, also adjusted for grade, gender, ethnic background and weight status of the child and educational level of the caregiver).

Table 3 also shows the unadjusted and adjusted results of the regression analyses. Given the unadjusted and adjusted results are very similar, only the results based on the fully adjusted model are described. Based on the ‘parent report’, no intervention effects were found for the outcome measure daily SSB consumption. Intervention effects were found on the outcome measure average SSB intake (SSB consumed in litres and number of SSB servings). Average SSB consumption in the intervention group was significantly lower than that in the control group (B -0.19 litres per day, 95% CI -0.28;-0.10, p < 0.001). The decrease in the number of SSB servings was also significantly higher in the intervention group than in the control group (B -0.54 servings per day, 95% CI -0.82;-0.26, p < 0.001). On the basis of the ‘child report’ we found no significant intervention effects for any of the outcome measures (p > 0.05 for daily SSB consumption and average SSB intake in litres or servings). On the basis of the ‘observation report’ , we found the increase in SSB brought to consume at school to be significantly smaller in the intervention group than in the control group (OR 0.51, 95% CI 0.36;0.72, p < 0.001).

When we evaluated interactions between condition and socio-demographic characteristics, child’s weight status at baseline or school pair, we found no significant results on the basis of the ‘parent report’ or ‘child report’ (p > 0.10). However, on the basis of the ‘observation report’, caregiver’s educational level and school pair appeared to be significant as effect modifiers (p < 0.10).

After conducting stratified analyses, we found no significant effect of the intervention for children of caregivers with a low educational level (high educational level OR 0.43, 95% CI 0.25;0.77, p = 0.004; and unknown educational level OR 0.45, 95% CI 0.22;0.91, p = 0.027).

The intervention effect was found only within one school pair (OR 0.37, 95% CI 0.22;0.64, p < 0.001). Regarding the other school pair, children at the intervention school did not differ significantly from the children at the control school with respect to bringing SSB to school.

## Discussion

This study evaluated the ‘water campaign’ programme. We found an effect on SSB on the basis of two of the three sources of information that were used to assess SSB consumption (i.e. ‘parent report’ and ‘observation report’).

Although the intervention had no effect on whether or not children consumed SSB on a daily basis, their average SSB consumption did change: after one year of intervention, on the basis of information gathered using the ‘parent report’, both average SSB consumption and average SSB servings were lower for children in the intervention group than for children in the control group. On the basis of information gathered using the ‘child report’, no significant differences in average SSB intake (in litres or servings) were found between children in the intervention and control group. An explanation for this discrepancy is lacking, but the lack of effect seen with the ‘child report’ can most likely be attributed to the fact that children are still too young to properly estimate their behaviour. Children’s inability to conceptualize – not only SSB but also the concepts of frequency and averaging – make it debatable whether these young children provide valid responses to food questionnaires that have items covering periods greater than one day [64]–[66]. In addition, research has shown that parents are more prone to reporting socially desirable answers compared to children [67]. This could also partly explain the fact that SSB consumption reported by children was higher than that reported by parents. On the basis of the ‘parent report’, no differences in intervention effect were found between the younger children (grades 2 to 4) and the older children (grades 5 to 7) (p > 0.05, data not shown). The parent-reported SSB consumption is probably more reliable and is supported by similar findings in the observations.

After one year of intervention, the number of children bringing SSB to school was lower in the intervention condition than in the control condition. Although the observations did not measure total daily SSB consumption, merely what children brought along to school for break time, they were the most objective measure of SSB consumption in our study. Furthermore, what children bring along to school is most probably largely dependent on their parents’ decisions.

The stratified analyses performed on the basis of the ‘observation report’ demonstrated that intervention effects are limited to subgroups. Differences in intervention effect were found between the two school pairs. Replication of the study with more clusters is recommended to confirm or reject our findings. Also, the effect of the intervention differed according to caregiver’s educational level in a manner that contradicted our expectations. Because the intervention schools are located in socially more deprived neighbourhoods, we expected to see an intervention effect among children of caregiver’s who have lower levels of education. This contradictory finding could be due to some degree of response bias: we may have had higher responses from caregivers with a higher level of education. It could also be explained by the large group of caregivers with an ‘unknown’ educational level that we found in the ‘observation report’.

A number of studies have been published on interventions that aimed to reduce SSB consumption by promoting water. These studies found similar but smaller intervention effects: for example, Tate et al. found a 80.7 ml decrease in SSB intake after a 6-month intervention and Sichieri et al. found a 55.0 ml SSB decrease after a one-year intervention [11],[13]. The study of Muckelbauer et al. found a significant increase in water consumption, but no effects on the consumption of juice or soft drinks were observed after adjustment for ethnic background and baseline intake [12]. Compared with these other studies the intervention effects in our study are thus encouraging.

Although the intervention was aimed at reducing the intake of children’s SSB consumption by promoting the intake of water, water consumption was not an outcome measure of our study. Despite this, we did explore the average intake of water, measured in litres, as reported in the parent and child questionnaires. On the basis of the ‘parent report’, there was a significant overall increase in water intake over time in both the intervention and control groups (respectively p < 0.001 and p = 0.015). However, on the basis of the ‘parent report’ and the ‘child report’ we found the intervention to have no effect on children’s water consumption (p > 0.05; see Additional file 1: Table S2). When we also explored whether the decrease in SSB consumption could be explained by an increase in water intake, we found that children with reduced SSB consumption did not differ in their water consumption at follow-up (p > 0.05; data not shown). These findings correspond with those of Veitch et al. [68]. However, since the mechanisms underlying the decrease in SSB consumption still remain unclear, further research is required.

The fact that we found an effect on SSB consumption does not necessarily imply a decrease in total energy intake or weight gain. However, a number of studies have indicated that a reduction in SSB consumption can have beneficial effects on total energy intake and weight status/BMI. For instance, Daniels and Popkin demonstrated that replacing SSB with water reduced total energy intake, implying less weight gain which may well contribute to preventing overweight [9]. In addition, the study by De Ruyter et al. demonstrated that replacing SSB with sugar-free alternatives resulted in reduced weight gain [7]. We explored the effects of the intervention on child’s BMI and weight status which are shown in Additional file 1: Table S3. Children in the intervention group had a significant higher increase of BMI compared to children in the control group (0.26BMI, 95% CI 0.11;0.40, p = 0.001). According the effect size criteria by Cohen, this can be regarded as a negligible effect (*d* = 0.03) [69].

The intervention in our study was a school- and community-targeted intervention, developed using social marketing. Our results suggest that a combined school and community approach may be beneficial for children to successfully develop healthier intake of drinks, supporting Bleich's et al. findings [35]. Furthermore, the use of social marketing meant that it was also possible to aim the intervention at a specific population (i.e. Turkish and Moroccan families) within a specific setting (i.e. socially more deprived neighbourhoods). However, when we explored whether such tailoring of the water campaign specifically to these minorities improved the effects seen among these children, we were unable to detect significant differences in intervention effect between children of Turkish and Moroccan background and children from other ethnic backgrounds (p > 0.05 in all three data sets; data not shown). However, the fact that the intervention had similar effects among all ethnic groups could be an indication that the reach and participation among this hard-to-reach target audience has improved, possibly due to the application of social marketing. We recommend that future studies should include a larger sample to increase the power for detecting behavioural changes within such a varied population.

### Strengths and limitations

The main strengths of this study are the setting and the duration (i.e. activities in daily practice at primary schools and in neighbourhoods for over a year). The study’s pragmatic setting means that the effects can be generalized to similar settings. A further strength of this study is that we used observations as well as questionnaires to determine the children’s SSB consumption.

A limitation of this study is the fact that randomization on the individual level was not possible. A further limitation is the small number of clusters (i.e. four), which inhibited multi-level analyses but was countered by adding the ‘school pair’ variable in the analyses. Since the use of self-report questionnaires to assess behavioural change is subject to limitations (e.g. misreporting of behaviour and providing socially desirable answers), we used different methods (i.e. observations and questionnaires) and assessed questionnaires from both parents and children. The non-response of parents to the parent questionnaire (complete case analyses only possible for 35%) is another limitation of this study. Our study included a diverse group of children with different ethnic backgrounds; between the three data reports the child’s ethnic backgrounds differed in distribution. Although no intervention effect of ethnic background and intervention condition was found, the intervention effects should be interpreted and generalized with caution (especially our findings based on the ‘parent report’). We assessed SSB intake ‘on average a day’ with the parent and child questionnaires and observed SSB consumption ‘on a random school day’. Further research is recommended to gain insight into different patterns of the child’s SSB consumption (e.g. on week-days vs. weekend-days). It may be debatable whether some beverages should be in- or excluded from the definition ‘SSB’. We recognize that some beverages may have additional nutritional benefits for children; however, we defined SSB in this study based on the amount of sugar within the beverages. A next step in altering the child’s consumption intake could be to give attention to and differentiate even more between SSB’s with and without nutritional value for the child’s diet. Finally, the water campaign consists of several components that promote water consumption. However, when applying such a multi-component intervention, it remains unclear which intervention activities are essential for obtaining the observed effects. We were unable to gather detailed implementation information as it was impossible to register the delivery of components at an individual level. Further research is therefore needed to understand the pathways of the behaviour changes that seem to have occurred.

## Conclusions

The findings of this study support the effectiveness of the water campaign in reducing the consumption of SSB, adding to evidence from other studies. Further studies are required to replicate the findings and to elucidate the possible mechanism underlying this intervention effect, the impact on BMI and the effectiveness in different subgroups. We also suggest that the water campaign be evaluated in other settings, using larger samples and with longer follow-up.

In the meantime, we recommend that schools and communities be aware of water as thirst-quencher, and as an alternative for SSB.

## Abbreviations

SSB: Sugar-sweetened beverages

BMI: Body mass index

EBF: Enjoy being fit

## Competing interests

All authors (VM van de Gaar, W Jansen, A van Grieken, GJJM Borsboom, S Kremers and H Raat) declare that they have no competing interests.

## Authors’ contributions

HR and WJ had the original idea for the study and its design, and were responsible for acquiring the study grant. VvdG further developed the study protocol and is responsible for data collection, data analysis and reporting the study results. GB assisted with the statistical analyses. HR and WJ supervise the study. WJ, AvG, SK and HR helped to refine the manuscript. All authors participated regularly in discussions regarding the design and protocols used in the study. All authors read and approved the final manuscript.

## Additional file

## Supplementary Material

Additional file 1: Figure S1. Response combinations for the different data reports. **Table S1.** Overview of items used to assess child’s SSB intake in the parent and child questionnaire. **Table S2.** Changes in water consumption in the intervention and control groups*. **Table S3.** Changes in BMI and weight status of the intervention and control groups*. Click here for file
