# Supplementary material for: Effects of an intervention aimed at reducing the intake of sugar-sweetened beverages in primary school children: a controlled trial
Source: Int J Behav Nutr Phys Act. 2014 Jul 25;11:98. doi: 10.1186/s12966-014-0098-8 (PMC4222660; doi:10.1186/s12966-014-0098-8)
Supplement: Additional file 1: Figure S1. — Response combinations for the different data reports. Table S1. Overview of items used to assess child’s SSB intake in the parent and child questionnaire. Table S2. Changes in water consumption in the intervention and control groups*. Table S3. Changes in BMI and weight status of the intervention and control groups*. [file s12966-014-0098-8-S1.docx]

Additional file

| **Table S1: Overview of items used to assess child’s SSB intake in the parent and child questionnaire** | | |
| --- | --- | --- |
| **Questionnaire items to assess child’s SSB intake** | **Response categories** | |
| *Introduction to questions related child’s SSB intake* | | |
| - Please indicate which of the drinks below your child (you) consume most of the times; this can be at school, at home or with friends. | - Coke/Pepsi - Fanta/Sisi - Fernandez - Dr. Pepper - Ice-tea - Energy drinks (Redbull etc) | - Lemonade - Apple juice - Yoghurt-drinks - Chocolate milk - Tea with sugar |
| *All questions below are related to so-called sugar-sweetened beverages (SSB). These are beverages containing added sugar, sweetened dairy products (e.g. chocolate milk), fruit juice (e.g. apple juice), soft drinks (e.g. cola) and energy drinks (e.g. sport energy drinks).*  *All of the above examples of drinks are SSB.*  *Please fill in the questions below on how much SSB your child/you consume and keep above described definition and examples in mind.*  *(So, do not take into account: light or sugar free beverages, water, 100% orange juice, tea without sugar, and regular milk.)* | | |
| - Does your child (do you) consume SSB on a daily basis? | - No, never - No, not every day - Yes, every day | |
| - Please indicate how many glasses (250ml – column A), cans (330ml – column B) or bottles (500ml – column C) the child (you) consumed on an average day on which the child drank SSB? |  | |

**
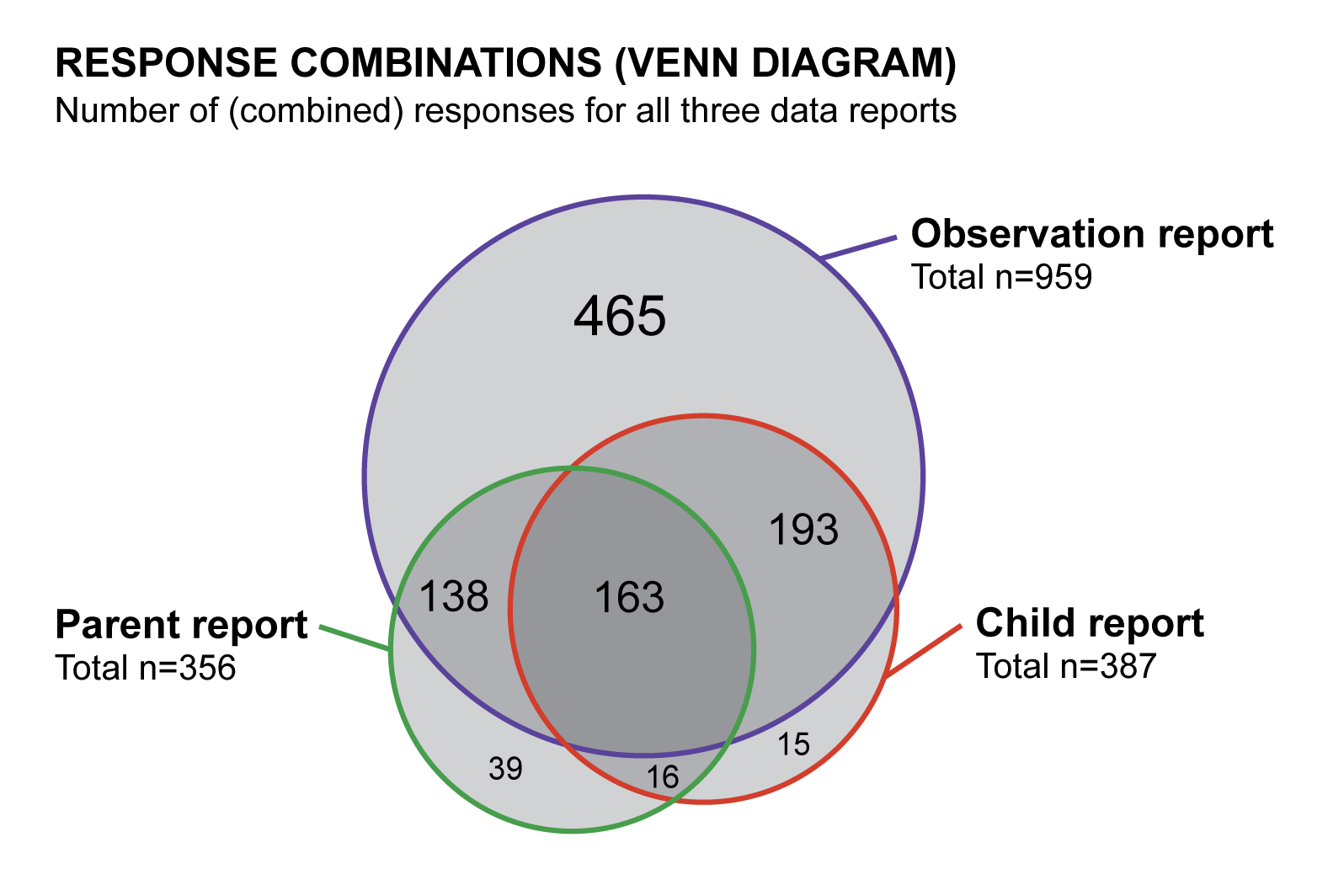
**

**Figure S1: Response combinations for the different data reports**

| **Table S2. Changes in water consumption in the intervention and control groups*** | | | | | | | | | | | | | | |  |  |
| --- | --- | --- | --- | --- | --- | --- | --- | --- | --- | --- | --- | --- | --- | --- | --- | --- |
|  |  | **Intervention** | |  | **Control** | |  | **Effect of intervention^c^** | | | | | | |  |  |
|  |  |  |  |  |  |  |  | **Unadjusted effects** | | |  | **Adjusted effects** | | |  |  |
| **Variable** | N | Baseline | Follow-up |  | Baseline | Follow-up |  | B | OR | 95% CI |  | B | OR | 95% CI |  |  |
| ***Parent report*** |  |  | |  |  | |  |  |  |  |  |  |  |  |  |  |
| Average water (L), mean (SD) | 350 | 0.59 (0.31) | 0.69 (0.33) |  | 0.62 (0.33) | 0.69 (0.33) |  | 0.02 |  | -0.04 - 0.08 |  | 0.03 |  | -0.03 - 0.09 |  |  |
| ***Child report*** |  |  | |  |  | |  |  |  |  |  |  |  |  |  |  |
| Average water (L), mean (SD) | 405 | 0.66 (0.29) | 0.67 (0.28) |  | 0.68 (0.28) | 0.66 (0.29) |  | 0.01 |  | -0.04 – 0.06 |  | 0.01 |  | -0.04 – 0.07 |  |  |
| Note: Mean values (SD) of average water intake in litres at baseline and follow-up, in intervention and control groups. Also shown is the intervention effect (B (95% CI) and OR (95% CI)) between both groups for the parent reports (n=350) and child reports (n=405). Significant effects are shown in **bold**. a = in favour of intervention group; b = difference (p<0.05) between intervention and control group at baseline; c = regression coefficients and odds ratios of the unadjusted models (only adjusted for baseline intake and school-pair) and fully adjusted models (in addition to baseline intake and school-pair, also adjusted for grade, gender, ethnic background and weight status of the child and educational level of the caregiver). | | | | | | | | | | | | | | |  |  |

| **Table S3. Changes in BMI and weight status of the intervention and control groups*** | | | | | | | | | | |  |  |  |  |
| --- | --- | --- | --- | --- | --- | --- | --- | --- | --- | --- | --- | --- | --- | --- |
|  |  | **Intervention** | |  | **Control** | |  | **Effect of intervention^c^** | | | | | | |
|  |  |  |  |  |  |  |  | **Unadjusted effects** | | |  | **Adjusted effects** | | |
| **Variable** | N | Baseline | Follow-up |  | Baseline | Follow-up |  | B | OR | 95% CI |  | B | OR | 95% CI |
|  |  |  |  |  |  |  |  |  |  |  |  |  |  |  |
| BMI, mean (SD) | 968 | 17.31 (3.02) | 18.10 (3.38) |  | 17.62 (3.06) | 18.24 (3.40) |  | **0.19** |  | **0.04 – 0.33** |  | **0.26** |  | **0.11 – 0.40** |
| Weight status, % overweight or obese | 968 | 22.1 % | 27.1 % |  | 24.4 % | 27.3 % |  |  | 1.27 | 0.80 – 2.02 |  |  | 1.27 | 0.78 – 2.08 |
| * = Mean values (SD) of child’s BMI and weight status at baseline and follow-up, in intervention and control groups. Also shown is the intervention effect (B (95% CI) and OR (95% CI)) between both groups for the children under study (n=968) with significant effects shown in **bold**. a = in favour of intervention group; b = difference (p<0.05) between intervention and control group at baseline; c = regression coefficients and odds ratios of the unadjusted models (only adjusted for baseline intake and school-pair) and fully adjusted models (in addition to baseline intake and school-pair, also adjusted for grade, gender, ethnic background and weight status of the child and educational level of the caregiver). | | | | | | | | | | | | | | |
